# Parental corporal punishment and adolescent drinking: the protective role of personal growth initiative and gender difference

**DOI:** 10.3389/fpsyg.2023.1199285

**Published:** 2024-01-08

**Authors:** Zhiyuan Tao, Zhenhai Wang, Mengyun Yin, Chengfu Yu, Wei Zhang, Haijun Dong

**Affiliations:** ^1^School of Psychology, South China Normal University, Guangzhou, China; ^2^School of Marxism, South China Normal University, Guangzhou, China; ^3^Key Laboratory of Brain, Cognition and Education Sciences (South China Normal University), Ministry of Education, Guangzhou, China; ^4^Center for Studies of Psychological Application and Guangdong Key Laboratory of Mental Health and Cognitive Science, South China Normal University, Guangzhou, China; ^5^Department of Psychology, Research Center of Adolescent Psychology and Behavior, School of Education, Guangzhou University, Guangzhou, China

**Keywords:** gender difference, parental corporal punishment, peer victimization, Chinese early adolescent drinking, personal growth initiative

## Abstract

**Introduction:**

Parenting and peer victimization (PV) are crucial for adolescent drinking. To further explore the cause of adolescent drinking, the present study investigated the role of PV and personal growth initiative (PGI) in the relationship between parental corporal punishment (PCP) and adolescent drinking.

**Methods:**

Present study build moderated mediation models to test the hypothesis, and detailed analysis of gender differences was conducted on the models. The data were collected in a cross-sectional questionnaire study with *n* = 1,007 adolescents (mean age = 13.16 years, 51.84% girls, *n* = 522).

**Results:**

Model analysis showed that: (1) PV totally mediated the relationship between PCP and adolescent girls’ drinking behavior; (2) The positive association between PV and drinking was only significant for girls with low PGI.

**Discussion:**

These findings underscore the importance of the protective effect of a personality trait characterized by spontaneous self-promotion on adolescent girls’ drinking.

## 1 Introduction

Alcohol consumption is a global health risk factor that affects both urban and rural populations, it should be responsible for numerous deaths and illnesses and leading to various alcohol-related harms and health problems and also result in billions of dollars in medical and social costs (GBD Oesophageal Cancer Collaborators, 2020; Friesen et al., 2022). Adolescence is a period of vulnerability during which youths may begin to exhibit harmful behaviors, such as drinking (Spear, 2018). Since the mid-2000s, youth drinking has been declining in many high-income countries (Vashishtha et al., 2021), however, as a developing country, China has many underdeveloped areas where alcohol-related issues may be more prevalent (Friesen et al., 2022). A meta-analysis conducted in 2016 found that the past-30-day alcohol use rate among Chinese middle schoolers was 19% (Feng and Newman, 2016), alcohol consumption in China is projected to continue increasing in the coming decade due to rapid economic growth and weak alcohol deterrence policies (World Health Organization, 2018). Taking into account the aforementioned factors, it is likely that adolescent drinking in China will become a significant social and public problem, and may be associated with various adverse behavioral and mental outcomes in adolescents (eg. risk of sexual behavior, interpersonal violence, criminality, depression and anxiety) (Woodin et al., 2016; Waterman et al., 2019; Lee and Feng, 2021). Except of the negative impact on adolescent health, there is also a certain possibility that alcohol use may affect the brain development of adolescent individuals, adolescent neurons are more vulnerable than adult neurons to the effects of alcohol, even after a single dose (Mira et al., 2020).

Parental corporal punishment (PCP) should be considered a significant environmental risk factor for adolescent drinking. PCP is defined as the use of physical force to cause children to experience pain, as a means of correcting or controlling their behavior (Straus and Stewart, 1999). It can include behaviors such as slapping, grabbing, and spanking children, and is sometimes used as a parenting strategy (Martin et al., 2020). Physical punishment by parents can lead to negative behavioral outcomes in children across many cultures, including the U.S., Korea, and China (Lee and Watson, 2020). In China, the traditional beliefs of “spare the rod and spoil the child” and “beating is an expression of love” have contributed to the acceptance of parental corporal punishment as a disciplinary mode by many parents, in order to motivate their children to achieve higher academic standards (Li et al., 2021). However, parental corporal punishment can result in negative behavioral and psychological outcomes for adolescents, including depression, aggression, stealing, and somatic complaints (Liu and Wang, 2018; Chen et al., 2021). Additionally, parents who accept corporal punishment during their children’s upbringing are more likely to engage in increasingly severe abusive behaviors (King et al., 2018).

Parental corporal punishment is considered a negative parenting practice and can lead adolescents to feel increased pressure and negative emotions (Chen et al., 2021). According to the self-medication theory (Cooper, 1994; Khantzian, 1997), adolescents may turn to alcohol to alleviate negative emotions and stress. Soloski (2020) asserted that self-medication is a common mechanism for concurrent drinking caused by negative events. Therefore, there may be a positive correlation between PCP and adolescent drinking (Pollard and McKinney, 2019). However, previous Chinese studies have reported conflicting findings regarding the association between PCP and adolescent drinking onset. One study using Cox proportional hazards modeling revealed a robust association between PCP in childhood and younger drinking onset during adolescence (Cheng et al., 2011) while another study using longitudinal data and logistic analysis did not find such effects of maternal corporal punishment (Alati et al., 2010). Given the potential public health risks associated with adolescent drinking and the conflicting findings in previous studies, an in-depth discussion on the potential mediating and protective mechanisms of PCP and early adolescent drinking is warranted.

### 1.1 Mediating effect of peer victimization

Peer victimization (PV), or repeated victimization by peers who are stronger in one or more aspects, can take many forms including direct physical victimization or threats, as well as indirect forms such as gossip, rejection, and verbal or relational victimization (Kawabata, 2020). The spillover theory posits that individuals are embedded in various interdependent social systems and changes in one system, such as the family environment, can affect individuals’ emotions and behavior in other systems, including the peer environment (Almeida et al., 1999; Ladd and Parke, 2021). It is worth noting that most previous studies have often used spillover theory to explain the spread of conflicts within family subsystems, and fewer studies have considered the spillover of conflicts across subsystems like this article. In recent years, researchers have begun to extend spillover theory to explain the mutual influence of family systems and peer systems, negative parenting, parental rejection, and peer victimization were proved that they can mutually reinforce each other through depression and anxiety (Kaufman et al., 2020). However, there is a slight lack of empirical research on conflict spillovers between subsystems. Only one research suggests that individuals who have suffered from PCP are more vulnerable to peer victimization and report more behavioral problems (Martin et al., 2020), whether PCP can affect PV still needs more exploration.

In turn, peer victimization has been identified as a risk factor for adolescent drinking (Fernandez et al., 2017). Self-medication theory is again helpful in explaining this phenomenon, as PV is a common source of stress in adolescence and is robustly associated with adolescent depression and anxiety (Liu et al., 2018). Drinking is also a strategy used by adolescents suffering from PV to cope with negative emotions, the experiences of victimization in cyberspace can also affect individuals’ psychological and behavioral health (Perasso et al., 2021a), overall, the harm of victimization experiences to adolescents cannot be ignored. Additionally, psychological needs and a sense of belonging are often compromised due to PV, leading some adolescents to engage in problematic alcohol use to compensate for their dissatisfaction and to release negative emotions (Rowe et al., 2019). PV has also been shown to indirectly increase the risk of drinking through a lack of school belonging in high school (Woerner et al., 2019). Therefore, we will investigate PV as a potential mediator and its differential effects on different gender.

### 1.2 The moderating effect of personal growth initiative

Although PCP may significantly contribute to adolescent drinking behavior via peer victimization, not all individuals are equally likely to use alcohol, there is an interaction between genetic factors and environmental factors, which can affect adolescent problem behavior. Personal growth initiative (PGI) is a spontaneous upward movement that makes people take initiative to develop themselves, consisting of four dimensions: being ready to change, planning, using resources, and intentional behavior, according to Robitschek’s research (2012). Adolescents with a high level of PGI will have a stronger ability and more positive strategies to cope with stressful situations, resulting in less drinking (Chen et al., 2020). This positive internal state will enable adolescents to have stronger resilience and be less likely to fall into deviant peer relations and behaviors (Yakunina et al., 2013), thereby improving their happiness and subjective well-being (Borowa et al., 2018). From a more macro perspective, fostering adolescent PGI could benefit public health by not only decreasing adolescents’ drinking but also reducing internet disorder, cyberbullying, problematic gambling (Loo et al., 2014; Gan et al., 2022), and further alleviate adolescent mental problems. Therefore, PGI was both importance for network health and public mental health.

However, while PGI has been extensively studied in the context of college and adult vocational development (Gong et al., 2022), its potential role in the context of peer victimization and adolescent drinking behavior has not been thoroughly investigated. Nevertheless, there is indirect evidence to suggest that high levels of PGI may act as a protective factor against adolescent drinking. Dowling et al. (2021) found that a combination of positive mental health characteristics, including PGI, emotional support, and autonomy, were associated with a reduction in delinquent behavior in adolescents. Moreover, a systematic review has shown that PGI is positively associated with psychological, social, and emotional well-being, as well as positive affect (Pinto Pizarro de Freitas et al., 2016). Taken together, individuals with high levels of PGI are more likely to report positive psychological states and actively engage in life, and as such, may be less prone to problematic drinking, even when faced with victimization (Chen et al., 2020). Thus, it is reasonable to hypothesize that PGI may play a role in mitigating the relationship between PV and adolescent drinking.

### 1.3 Gender difference

Gender differences in drinking behaviors among early adolescents in China are significant, with drinking being more socially acceptable for boys than girls. According to traditional Confucian values, women were discouraged from drinking because it is incompatible with traditionally feminine traits, women have been taught “Hyun-sook” (i.e., be gentle and virtuous, and asked to teach children and serve the husband at home) and join less social affairs from an early age (Cui et al., 2018). As a result, girls are often socialized to participate in fewer social activities that involve alcohol. Additionally, females tend to have a higher risk perception of drinking and engage in less risky drinking patterns in China compared to males (He et al., 2016). Previous research on Chinese adolescents has consistently found that boys report more drinking than girls (Bo and Jaccard, 2020). However, apart from cultural factors, girls may also be more susceptible to negative family influences, which could contribute to their increased risk of drinking (Soloski and Berryhill, 2015). At the same time, a review for a western country found that over time, the drinking rate of girls has gradually increased in the past 30 years and surpassed boys which is not the same as the situation in China, this phenomenon also indicates that researchers around the world still need to take a deeper discussion on the gender differences in adolescent alcohol consumption to understand the role of gender (Raninen et al., 2023).

It is important to consider gender differences in the relationship between PV, PGI, and adolescent drinking behavior. Previous research has shown that peer victimization has more negative effects on predicting earlier drinking onset among adolescent girls than boys (Kim et al., 2019; Boyd et al., 2020). Woerner et al. (2019) also believe that girls are more vulnerable to peer victimization than boys. Furthermore, while high PGI has been found to be associated with less drinking among college girls (Coleman et al., 2016). Although the above-mentioned relationships may not hold true for adolescent, it is important to investigate the potential gender differences in the relationship between PV, PGI, and adolescent drinking behavior in our model.

### 1.4 Aims and hypotheses

**Figure 1:**
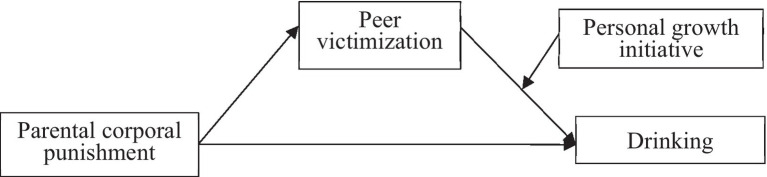
Moderated mediation model.

The aim of present study is test the potential mechanism of PCP and adolescent drinking, and we separately test boys and girls by the moderated mediation models. In sum, we proposed and tested a moderated mediation model (Figure 1) and test three hypotheses.

*H1:* PCP will positively predict adolescent drinking behavior: adolescents who experienced high PCP will show more drinking behavior.

*H2:* PV will significantly mediate the relationship between PCP and adolescent drinking.

*H3*: PGI will significantly moderate the pathway from PV to adolescent drinking. Specifically, the pathways from PCP and PV to drinking will be stronger among adolescents with low PGI.

## 2 Methods

### 2.1 Participants

In this study, *n* = 1,007 students from several public junior high schools in south China were recruited as subjects through convenience sampling. Among them, 485 were male and 522 were females (51.84% girls). The mean age of the participants was 13.16 years and the ages range ranged from 11.58 to 16.17 years (*SD* = 0.67 years).

### 2.2 Measures

#### 2.2.1 Parental corporal punishment

The Parental Corporal Punishment Questionnaire (Xu et al., 2017) was used. Participants were asked to report the number of times they had suffered corporal punishment from their parents, such as slaps/spanking, shaking, and other punishments in the past 6 months, there are 5 items on the scale. Each item is rated on a five-point scale, with 1 being “never” and 5 being “six times or more” and the total score being the average score for all items. The higher the score, the more corporal punishment there was. In the present study, Cronbach’s α of the questionnaire was 0.88. The questionnaire show great reliability and validity in the past research (Zhu et al., 2019).

#### 2.2.2 Peer victimization

Peer victimization was measured by the questionnaire used by Zhou et al. (2014). The questionnaire consisted of 9 items, and participants were asked to self-report the number of times they had experienced peer victimization in the past 6 months, such as “Have you ever been scolded by a mate,” and “Have you ever been discriminated against by your peers.” A five-point scale was used, with 1 representing “never” and 5 representing “four times or more.” Finally, the average score of the 9 items was calculated. The higher the average score, the more frequent peer victimization. In this study, the Cronbach’s α of this questionnaire was 0.94. The questionnaire show great reliability and validity in the past research (Li et al., 2022).

#### 2.2.3 Personal growth initiative

PGI was assessed by the second edition of the Personal Growth Initiative Scale (PGI-II; Robitschek et al., 2012) and translated by Guo and Ye (2016) The PGI-II has 4 factors: readiness for change (e.g., I know the aspect I need to change), planning(e.g., I know how to make a plan to change myself), utilization of available resources (e.g., I will ask someone for help when I met some trouble in my growing process), and initiative (e.g., I can catch every chance that makes me stronger), which were subdivided into 16 statements. Responses were on a 5-point Likert scale ranging from 1 = “very inconsistent” and 5 = “very consistent.” The higher the total score, the higher the personal growth initiative. In this study, the measure demonstrated good reliability, Cronbach’s α was 0.95 in the present measurement. The questionnaire show great reliability and validity in the past research (Chen et al., 2020).

#### 2.2.4 Drinking behavior

Participants reported the average number of times per month they had used alcohol (including beer, wine, and hard liquor) in the past 6 months. Responses were on a 6-point Likert scale from 1 = never to 6 = 8 or more times (Shigemoto and Robitschek, 2018). Higher scores indicated higher levels of drinking behavior. This instrument has demonstrated good validity in previous studies (Jiang et al., 2015). Higher scores mean a higher level of adolescent alcohol use. The Cronbach’s α was 0.92 for this questionnaire in the study. The questionnaire show great reliability and validity in the past research (Wang et al., 2022).

### 2.3 Procedure

This research was conducted with permission from the Academic Ethics Review at the university with which the second author is affiliated, we used the convenient sampling and conducted sampling at a middle school in Guangdong Province. Before beginning the investigation, all the parents and students provided informed consent for their adolescents to participate. At the beginning of the data collection session, it was emphasized to the participants that their answers to the questionnaires would be anonymous, questionnaires would be immediately sealed safekeeping, and only used for academic research. Adolescents were then instructed to complete the questionnaires independently. Data collection took place in the students’ regular classrooms during class time. The completion time was less than 30 min. Samples with a response time of less than 10 min or too many missing values will be screened out. After determining the available samples, we used the mean imputation method to deal with the missing values.

### 2.4 Statistical analyses

First, we used SPSS 26.0 to inspect descriptive statistics and Pearson correlations among variables and standardize the variables. Next, Model 4 of the PROCESS 4.0 macro was used to evaluate the mediation model and test the total effect of PCP on adolescent drinking, and mediating effects of PV. The final step used Model 14 to explore the moderated mediation model. These tests were conducted using bootstrapping with 5,000 iterations. Finally, a Johnson-Neyman test was used to investigate the more detailed effect of PGI.

## 3 Results

### 3.1 Mean, standard deviation, and correlation coefficient of each variable

PCP was significantly positive correlated with adolescent drinking (*r* = 0.08, *p* = 0.02) and PV (*r* = 0.37, *p* < 0.001), and PV was significantly positive related to drinking (*r* = 0.14, *p* < 0.001). PGI was significantly negative correlated to PV (*r* = −0.13, *p* < 0.001) and drinking (*r* = −0.11, *p* < 0.001, in Table 1). The correlation coefficient between gender and PV (*r* = 0.08, *p* = 0.01) shows that boys report more PV. These findings suggest that it is warranted to test the mediating effect of peer victimization and the moderating effect of a growth initiative in the moderated mediation model for different genders. And we test the common method bias was assessed by the Harman one-way test before model construction, the load of the maximum factor was 31.59%, which is less than the critical value of 40% (Lee et al., 2011).

**Table 1:** Means, standard deviations, and Pearson correlation coefficients of all variables.

Variables	1	2	3	4	5	6
1. Gender	-					
2. Age	0.06	–				
3. PCP	0.03	−0.09**	–			
4. PGI	0.02	−0.02	−0.14***	–		
5.PV	0.08*	−0.04	0.37***	−0.13***	–	
6.Drinking	−0.02	0.05	0.08*	−0.11***	0.14***	–
Mean (range)	0.48 (0–1)	13.16 (11.58–16.17)	1.14 (1–4)	3.54 (1–5)	1.48 (1–5)	1.07 (1–4)
*SD*	0.50	0.67	0.35	0.73	0.60	0.30

Gender is a dummy variable, *n* = 1,007; 1 = boy, 0 = girl; PCP, parental corporal punishment; PV, peer victimization; PGI, personal growth initiative. *p < 0.05; **p < 0.01; ***p < 0.001.Bold values are significant values.

### 3.2 Moderated mediation model test

The moderated mediation model was constructed in the total sample first, PCP could positive predict PV [*β* = 0.37, *t* = 12.54, *p* < 0.001, 95% CI (0.31, 0.43)], and PV positive predict adolescent drinking (*β* = 0.13, *t* = 3.74, *p* < 0.001, 95% CI [0.06, 0.19]), the residual effect of PCP on drinking was not significant [*β* = 0.01, *t* = 0.27, *p* = 0.79, 95% CI (−0.06, 0.08), in Table 2]. PGI could significantly moderate the pathway from PV to drinking [*β* = −0.06, *t* = −2.62, *p* < 0.01, 95% CI (−0.11, −0.02)], specifically, the indirect effect (IE) was not significant in the high PGI adolescents but significant in the low PGI adolescents [*IE of high PGI* = 0.02, *SE* = 0.01, 95% CI (−0.004, 0.05), *IE of low PGI* = 0.07, *SE* = 0.03, 95% CI (0.02, 0.14)].

**Table 2:** Model results of the total sample.

Predict Variables	Equation 1: PV			Equation 2: Drinking		
	*β*	*SE*	*t*	*β*	*SE*	*t*
Gender	**0.14**	0.06	−2.40*	−0.06	0.06	−0.89
Age	−0.01	0.03	−0.37	0.06	0.03	1.90
PCP	**0.37**	0.03	12.54***	0.01	0.03	0.27
ME: PV				**0.13**	0.03	3.74***
MO: PGI				**−0.08**	0.03	−2.66**
ME × MO				**−0.06**	0.03	−2.62**
Indirect effect (MO_Mean - 1SD_)		*Effect size of low PGI* = 0.02, 95% CI [−0.004, 0.05]				
Indirect effect (MO_Mean + 1SD_)		*Effect size of high PGI* = 0.07, 95% CI [0.02, 0.14]				
***R***^***2***^	0.14			0.04		
***F***	55.73***			6.81^***^		

Gender is a dummy variable, *n* = 1,007; 1 = boy; 0 = girl; PCP, parental corporal punishment; PV, peer victimization; PGI, personal growth initiative; ME, mediating variable; MO, moderating variable. *p < 0.05; **p < 0.01; ***p < 0.001. Bold values are significant values.

Then we build the mediation models for different genders, we found that the moderated mediation model was only established for girls. Among boys, the moderated mediation model was not established, the indirect effect was not significant [*IE* = 0.01, *SE* = 0.01, 95% CI (−0.01, 0.04)], and the direct pathways of PCP [*β* = 0.03, *t* = 0.61, *p* = 0.54, 95% CI (−0.06, 0.10)] and PV on drinking [*β* = 0.03, *t* = 0.78, *p* = 0.44, 95% CI (−0.05, 0.12)] were not significant too. Besides, PGI could not moderate the pathway from PV to adolescent boys’ drinking [*β* = −0.01, *t* = −0.40, *p* = 0.69, 95% CI (−0.07, 0.05)].

**Figure 2:**
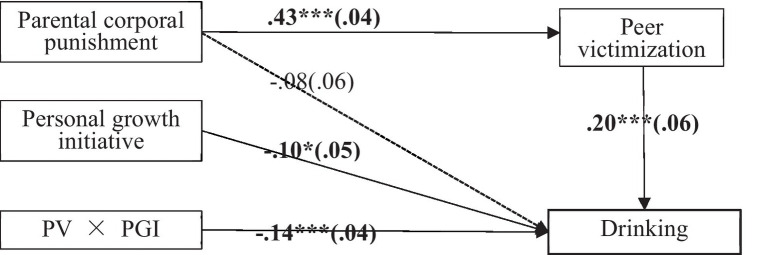
Results of moderated mediation modern of girls. Note: Values outside the brackets are standardized regressive coefficients, *n* = 522, values in the brackets are standardized SE. *p < 0.05, ***p < 0.001. Moderated mediating effect tested by the bootstrap method: (1) Among girls with low PGI (Mean - 1SD), the indirect effect was significant [IE = 0.16, SE = 0.07, 95% CI (0.05, 0.31)]; (2) Among girls with medium PGI (Mean), the indirect effect was significant [IE = 0.10, SE = 0.04, 95% CI (0.03, 0.19)]; (3) Among girls with high PGI (Mean + 1SD), the indirect effect was insignificant [IE = 0.05, SE = 0.04, 95% CI (−0.04, 0.13)].

Among girls, PCP positively predicted girls’ PV [*β* = 0.43, *t* = 10.28, *p* < 0.001, 95% CI (0.34, 0.51), in Figure 2], and PV positively predicted adolescent girls’ drinking [*β* = 0.24, *t* = 4.48, *p* < 0.001, 95% CI (0.13, 0.34)], and the residual direct effect of PCP on drinking was not significant [*β* = −0.05, *t* = −0.96, *p* = 0.34, 95% CI (−0.17, 0.06)]. The indirect effect of PCP on adolescent girls’ drinking through PV was significant [*IE* = 0.10, *SE* = 0.04, 95% CI (0.03, 0.19)], which indicate that PV totally mediates the relationship between PCP and girls’ drinking. PGI significantly moderated the direct pathway of PV to drinking [*β* = −0.13, *t* = −3.48, *p* < 0.001, 95% CI (−0.21, −0.06)] and the mediating pathway. Further, the bootstrap method was used to test the significance of the moderated mediating effect: (1) Among girls with low PGI, the indirect effect of the mediation pathway was significant [*IE* = 0.16, *SE* = 0.07, 95% CI (0.05, 0.31)]. (2) The *IE* was not significant among high PGI girls [*IE* = 0.05, *SE* = 0.04, 95%CI (−0.04, 0.13)].

**Figure 3:**
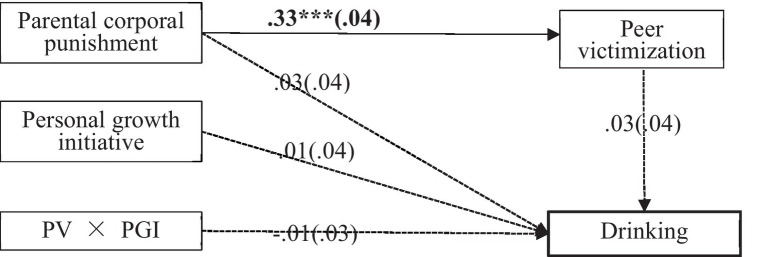
Results of moderated mediation model for boys. Note: Values outside the brackets are standardized regressive coefficients, *n* = 485, values in the brackets are standardized SE. ***p < 0.001.Moderated mediating effect tested by the bootstrap method: (1) Among boys with low PGI (Mean - 1SD), the indirect effect (IE) was insignificant [IE = 0.01, SE = 0.02, 95% CI (−0.01, 0.05)]; (2) Among boys with medium PGI (Mean), the indirect effect was insignificant [IE = 0.01, SE = 0.01, 95% CI (−0.01, 0.04)]; (3) Among boys with high PGI (Mean + 1SD), the indirect effect was insignificant [IE = 0.01, SE = 0.01, 95% CI (−0.01, 0.03)].

To explain the interaction in girls more clearly, simple slopes tests were carried out. Specifically, the pathway of PV to drinking was calculated when the mean growth initiative was plus (high PGI) or minus (low PGI) one standard deviation (Mean ± 1*SD*, in Figure 3). Results for girls showed that: (1) Among girls with low PGI, PV was significantly positively predicted drinking [*β* = 0.38, *t* = 5.81, *p* < 0.001, 95%CI = (0.22, 0.50)]. (2) There was no significant correlation between PV and drinking among girls with high PGI [*β* = 0.11, *t* = 1.62, *p* = 0.11, 95%CI = (−0.02, 0.24)].

**Figure 4:**
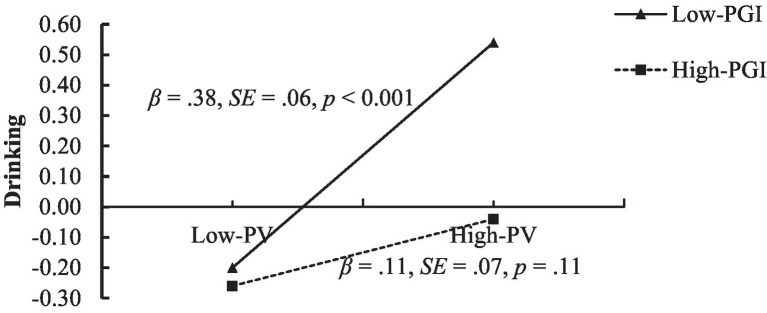
The simple slopes test results of the moderating effect of girls.

And we made a more detailed Johnson-Neyman test which was called the “spotlight test” and could search for the significant point of simple slopes and the corresponding *Z* value of the moderator (Spiller et al., 2013), results in Figure 4 showed the relationship between PV and adolescent girls’ drinking was significant while the *Z*-score of PGI was lower than 0.85, and girls with *Z*-score of PGI below 0.85 accounts for 84.48% which highlight the importance of PGI for girls.

## 4 Discussion

**Figure 5:**
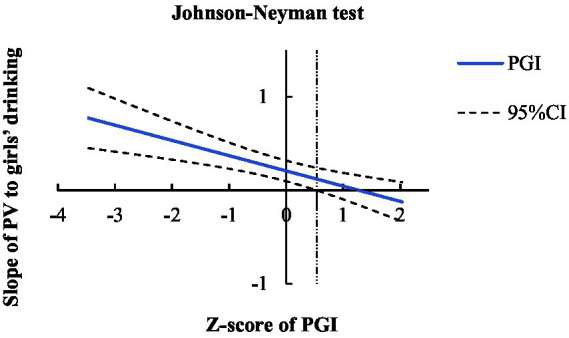
Johnson-Neyman test results of girls. The solid blue line shows the change trend of slopes of PV to girls’ drinking while PGI increasing, and coarse dotted lines show the 95%CI of each slopes and the vertical thin dotted line was the dividing line between significance and insignificance of the slopes. While the *Z*-score of PGI was lower than 0.85, the relationship between PV and drinking was significant among adolescent girls, *n* = 522.

Adolescent drinking is a prevalent and risky behavior, it impacts adolescent brains’ development (e.g., accelerated decline of gray matter) and could influence adolescents’ long-term development (de Goede et al., 2021), it increases alcohol related problems to put a burden on social economic and public health. Therefore, our results have important implications for revealing a potential path of PV between PCP and middle school students’ drinking and the protection role of PGI. We found a significant positive correlation between PCP and adolescent drinking, this pattern of results indeed showed the concurrency between PCP and drinking, and the missing residual effect in the mediation model emphasized the importance of PV, according to the self-medication theory, adolescents who have experienced peer bullying will try drinking alcohol to alleviate the negative impact of this interpersonal experience (Figure 5).

The model results demonstrate that the relationship between PCP and adolescent girls’ drinking was completely mediated by PV, providing partial support for our hypothesis. Spillover theory (Ladd and Parke, 2021) and prior research have suggested that children who experience negative parenting, such as PCP or parental rejection, in the family environment are also at risk of victimization in other contexts (Kaufman et al., 2020). This spillover effect can be explained by several factors. Firstly, adolescents who undergo corporal punishment in the family system may exhibit symptoms of maladjustment (e.g., anxiety, depression, conduct problems, social withdrawal), which can put them at a disadvantage and make them more susceptible to self-blame, thereby increasing their vulnerability to peer victimization (Kaufman et al., 2020). Secondly, PCP as a form of family violence can lead to a perpetuating cycle of violence for adolescents in the family or other social systems (e.g., school), causing them to adopt violent norms and potentially develop learned violence while socializing with more violent peers, ultimately heightening their risk of peer victimization (Xia et al., 2018). And our results are helpful in promoting the combination of self-medication theory and spillover theory, specifically, adolescent who experience any type of interpersonal violence are likely to suffer from other types of bullying. Drinking may be a refuge for them to avoid these negative emotions caused by the victimized experience, this indicates that alcohol use may be a non adaptive treatment method for negative interpersonal experiences. This may be a good beginning to propose interpersonal-medication theory. After clarifying the negative effects of PCP, it is particularly important to suggest that parents adopt a positive parenting approach and parental control was proved that it could be a protective factor reduced the frequency of adolescent drinking (Perasso et al., 2021b).

The positive association between PV and drinking was significant and consistent with previous studies. Adolescents may use alcohol to cope with negative emotions caused by PV, such as anxiety and hopelessness (Soloski, 2020). Additionally, PV may cause adolescents to excessively focus on their internal state, leading to increased alcohol use as a coping strategy (Meisel et al., 2018). Furthermore, PV may lead adolescents to accept violent norms, increasing their risk of associating with deviant peers and conforming to social drinking norms (Pesola et al., 2015). Boys, on the other hand, may be more likely to fight back against peer victimization but not endure the pain in silence. Thus, while the two segments of mediating pathways in boys were significant, we did not observe a mediating effect of PV on boys’ drinking.

### 4.1 Protective effect of personal growth initiative on adolescent girls

The results of the study revealed that PGI only had a moderating effect on adolescent girls, girls who had high PGI exhibiting higher levels of self-initiative and autonomy in avoiding deviant behavior such as drinking. The various components of PGI, including self-respect and respect for others, can reduce drinking frequency by improving self-esteem, and greatly reduce the negative impact of PV (Pesola et al., 2015; Pinto Pizarro de Freitas et al., 2016). Those girls with low PGI are unable to regulate their behaviors, while experienced high levels of peer victimization, they may not able to handle their negative emotions (e.g., anxiety and depression) (Woerner et al., 2019), and may be more likely to engage in drinking behaviors. At the same time, such girls may lack internal drive to positively plan for the future and improve themselves, and they may be more reluctant to devote their energies to positive behavior. When they are unable to deal with PV experience, they also lack the ability to use adaptive solutions. Furthermore, it is important to note that although we have found a full mediating effect in girls, we do not deny that there may be other mediating variables that can explain the relationship between PCP and girls’ drinking. More research is needed to analyze the underlying mechanism of this relationship.

Adolescent girls are more vulnerable to developing alcohol use disorder (AUD) than boys. While research conducted in another Asian country (i.e., Korea) has shown that more adolescent boys are drinkers than girls, adolescent girls who do drink exhibit higher rates of risky drinking behavior than boys (Jeon et al., 2020). Furthermore, adolescent girls are more likely to experience stressful life events, and are more susceptible to neurodevelopmental deficits, which can impair attention performance and lead to thinner frontal cortical development in girls with drinking disorders as compared to boys. Continued alcohol use by adolescent girls puts them at higher risk for brain damage and behavioral problems in the future. The impact of drinking on adolescent girls’ development is significant and has been linked to various public health problems, such as anxiety, risky sexual decision-making, and drug use (Woodin et al., 2016; Waterman et al., 2019; Lee and Feng, 2021). Our results suggest that cultivating adolescent girls’ PGI may be a possible intervention for addressing these issues.

According to traditional values (Cui et al., 2018), avoiding alcohol is not considered a positive behavior for boys and could even be viewed as damaging to their masculinity which can explain the insignificance effect of PGI on boys (Piko, 2006). Conversely, drinking is viewed as a violation of gender norms for girls (Cui et al., 2018), and as such, high PGI girls may be more likely to control their behaviors to comply with gender norms avoid engaging in drinking, they are more likely to devote their energy to planning the future and mobilizing resources, and are more able to respond to negative environments in a positive manner.

Although PGI only shows a protective effect for girls, the value of PGI has not affected by this partially protective effect. It highlights the importance of cultivating girls’ initiative to seek self-improve. Furthermore, our Johnson-Neyman test showed that the majority of girls (84.48%) have not developed sufficient PGI, placing them at risk of engaging in drinking behavior. We are also concerned that they may face other psychological and behavioral challenges in the future. Implementing short-term self-growth goals and programs for these adolescents can be a proven and effective intervention (Shigemoto, 2021). For early adolescent girls, setting attainable goals related to academic achievement, physical exercise, moral education, and aesthetic education may be a reasonable plan to improve their PGI, reduce the negative consequences of drinking, and alleviate the burden of public health.

### 4.2 Implications and limitations

The results of this study contribute to the theoretical value of self-medication theory and spillover theory in the field of adolescent psychology, particularly in the context of families and schools. Our study specifically investigated the direct relationship between PCP and adolescent drinking, and found that PV played a crucial role in mediating this relationship among adolescent girls. Moreover, our findings also highlight the developmental importance of PGI in adolescent girls, as a protective factor against alcohol use disorder.

The implications of our study suggest that interventions targeting peer victimization as a risk factor for alcohol use should focus on increasing PGI in adolescent girls. In addition, future research should take into account gender differences when examining the relationship between drinking, PV, and PGI. It is also recommended that further research investigate other positive personality traits that may be beneficial for reducing drinking behavior among adolescent boys. It is concerning that the drinking rate among Chinese teenagers has been gradually increasing from 2002 to 2016, and this trend may continue in the future (Jeon et al., 2020). Therefore, we hope that our study can serve as a basis for the cultivation of positive psychological qualities (e.g., PGI) in teenagers, which may help to mitigate their drinking behavior and improve their overall well-being.

Although this study is valuable, there are some limitations that need to be addressed. First, it would be beneficial to use a range of research methods to collect more comprehensive data on other positive psychological characteristics or states (e.g., hope, gratitude) as buffers, and control for family and school-related variables more effectively. Second, the data were collected from a low-risk sample with lower reported drinking rates, so these findings may not generalize to high-risk groups. Third, as puberty is a unique period of development, the results may not be applicable to other age groups, and caution should be exercised when generalizing these findings. Lastly, using a longitudinal and cross-lagged model with multiple informants (e.g., teachers, parents, and peers) to confirm the relationships among these variables would be more effective than relying solely on cross-sectional research. Last but not least, solely self-report scales that may provoke social desirability in the respondents, using a multi-reporter and quali-quantitative methodology approach may avoid these defect.

### 4.3 Conclusion

The current study offers valuable insights into the relationship between PCP, PV, and alcohol use in girls, using a well-fitting moderated mediation model. The results suggest that peer victimization plays a significant mediating role in the relationship between PCP and drinking, and that girls with higher levels of PGI exhibit lower levels of drinking. These findings highlight the importance of encouraging early adolescent girls to cultivate their own personal growth, as it may help protect them against drinking behavior in the context of PV.

## Data availability statement

The original contributions presented in the study are included in the article/supplementary material, further inquiries can be directed to the corresponding author/s.

## Ethics statement

The studies involving humans were approved by the Ethics in Human Research Committee of the Department of Psychology. The studies were conducted in accordance with the local legislation and institutional requirements. Written informed consent for participation in this study was provided by the participants’ legal guardians/next of kin.

## Author contributions

CY, ZT, and ZW were performed the material preparation, data collection, and analysis. ZT wrote the first draft of the manuscript. All authors contributed to the study conception, design, commented on previous versions of the manuscript, read, and approved the final manuscript.
